# An unusual intraorbital foreign body: A brake lever

**DOI:** 10.4103/0301-4738.55063

**Published:** 2009

**Authors:** Mohammad Hosein Nowroozzadeh

**Affiliations:** Department of Ophthalmology, Khalili Hospital, Shiraz University of Medical Sciences, Shiraz, Iran

**Keywords:** Brake lever, intraorbital foreign body, management, orbital trauma

## Abstract

Orbital trauma usually affects the bony parts of the orbit; however, in rare cases foreign bodies are found within the orbit. In this report, we introduce a case with unusual large intraorbital foreign bodies (two parts of a brake lever) after a motorcycle accident. Although one of the foreign bodies was located in the posterior orbit, they required only one simple operation for retrieval. We will discus the management strategy.

Intraorbital foreign bodies (FBs) usually occur after a high-velocity injury such as a gunshot or industrial accident.[1,2] Although motorcycle accidents are a major cause of ocular and orbital trauma in Iran, intraorbital FB during these accidents is rare.

Traumatic eye injuries due to large foreign bodies are rare. There are few reports of unusually large intraorbital FBs such as an iron nut or wood.[3,4] There is controversy regarding the best management of intraorbital FBs. In this report, we present the clinical features and management of our patient initially seen with a large intraorbital FB.

## Case Report

**Figure 1:**
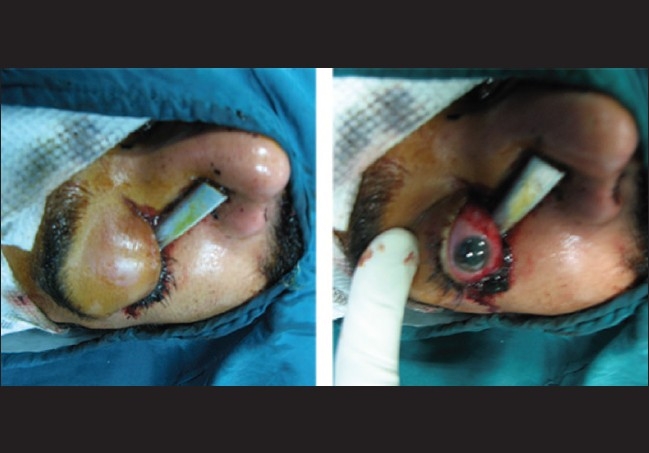
Penetrating intraorbital injury with brake lever

**Figure 2:**
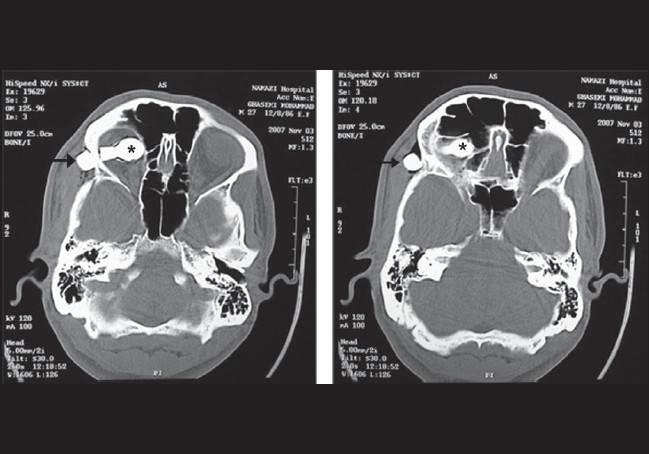
Orbital computed tomography shows two parts of foreign body: The short part (asterisk) located in the superior orbit, and the tip of the long part (arrow)

**Figure 3:**
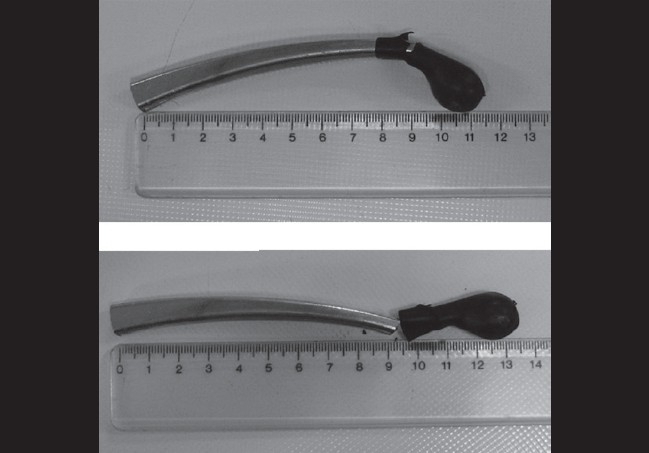
The brake lever after surgical removal

A 25-year-old man presented to our emergency clinic 6 h after a penetrating orbital injury with a brake lever in his right lower eyelid during a motorcycle accident [Fig. 1]. Both eyelids were echymotic and edematous. On examination, he had no light perception in the affected eye and 20/20 in the fellow eye. The left eye was otherwise normal. In alternating light test, the right pupil was completely redilated (complete right afferent pupillary defect). Slit-lamp examination and fundoscopy could not be performed on the right eye due to patient discomfort. Computed tomography (CT) revealed two pieces of metallic intraorbital FBs, one of which was embedded in the zygomatic bone, and the second one located in posterior superior orbit [Fig. 2]. A neurological examination showed no neurological deficits or signs of cranial penetration. With the impression of compressive optic neuropathy, we started intravenous methyl prednisolone and scheduled the patient for an emergency operation. With the patient under general anesthesia, the first portion of the metallic foreign body was removed from the lower eyelid with controlled and slow motions. On peritomy, we detected no corneal or scleral perforation. The inferior rectus muscle was disrupted. So, the anesthesiologist was informed of the situation and it was ensured that the anesthesia was deep. Then, we requested a zoom operating microscope, gently displaced the eye superiorly, retracted the Tenon's capsule from the globe with a Desmarres lid retractor (11 mm wide, Storz, E-989), and the area of Tenon's capsule where the muscle was expected to penetrate was inspected. Although the second part of the foreign body was located in the superior posterior orbit and its removal seemed to be difficult, we could fortunately find and remove it without significant resistance during exploration for the lost inferior rectus muscle via its tract in the inferior orbit [Fig. 3]. All bleeding sites were touched up with light cautery and we continued to search for the lost muscle. Eventually, we could find the lost muscle and grasped it with a two-arm 5-0 vicryl suture. Then the suture was passed through the muscle stump at the insertion site and was tied. Conjunctiva and eyelids were sutured with proper materials. Four weeks after the surgery, the patient had no light perception, complete ptosis and a frozen eye due to multiple cranial nerve injury. Ocular examination showed normal anterior and posterior segments. The only positive findings were complete afferent papillary defect and optic atrophy in the right eye.

## Discussion

Intraorbital FBs can be associated with severe injuries leading to loss of vision or may lead to sight-threatening complications.[1,2] A retained metallic intraorbital FB may cause a variety of signs, symptoms, and clinical findings, based on its size, location, and composition.[5] Loss of vision is usually due to the initial trauma and is generally not influenced by surgical intervention.[1] The best management of retained metallic intraorbital FBs remains a controversial subject.[5,6] The decision regarding surgical removal depends mainly on the location and type of intraorbital FBs.[5] However, the removal of foreign body from the orbit, which is crowded with delicate structures, is not safe.[6]

Retained metallic intraorbital FBs are well tolerated and should be managed conservatively in the absence of specific indications for removal.[1,2] When the foreign body is impinging on neurological structures or causing mechanical restriction to ocular movements or is composed primarily of copper, one should consider removal of the FB after detailed and precise localization to minimize damage to the adjacent ocular structures.[1,2,5,6] In this patient because of the supposed compressive optic neuropathy, we decided to remove the intraorbital FBs. Although the second part of the FB seemed to need lateral orbitotomy approach for removal, to our astonishment we could remove it via its tract in the inferior orbit with an anterior surgical approach. This experience shows that some intraorbital FBs especially those with round and smooth surfaces can simply be removed from their tract, obviating the need for more sophisticated surgery. This approach may especially be useful in large intraorbital FBs in which the tract is easily discernible and removal of foreign body with other approaches may render significant trauma to frail orbital structures.
